# A Multi-Stage *Plasmodium vivax* Malaria Vaccine Candidate Able to Induce Long-Lived Antibody Responses Against Blood Stage Parasites and Robust Transmission-Blocking Activity

**DOI:** 10.3389/fcimb.2019.00135

**Published:** 2019-05-01

**Authors:** Jessica N. McCaffery, Jairo A. Fonseca, Balwan Singh, Monica Cabrera-Mora, Caitlin Bohannon, Joshy Jacob, Myriam Arévalo-Herrera, Alberto Moreno

**Affiliations:** ^1^Emory Vaccine Center, Yerkes National Primate Research Center, Emory University, Atlanta, GA, United States; ^2^Division of Infectious Diseases, Department of Medicine, Emory University, Atlanta, GA, United States; ^3^Department of Microbiology and Immunology, Emory University School of Medicine, Atlanta, GA, United States; ^4^Caucaseco Scientific Research Center, Malaria Vaccine and Drug Development Center, Cali, Colombia

**Keywords:** malaria, *Plasmodium vivax*, multi-stage, chimeric vaccine, transmission-blocking, P25

## Abstract

Malaria control and interventions including long-lasting insecticide-treated nets, indoor residual spraying, and intermittent preventative treatment in pregnancy have resulted in a significant reduction in the number of *Plasmodium falciparum* cases. Considerable efforts have been devoted to *P. falciparum* vaccines development with much less to *P. vivax*. Transmission-blocking vaccines, which can elicit antibodies targeting *Plasmodium* antigens expressed during sexual stage development and interrupt transmission, offer an alternative strategy to achieve malaria control. The post-fertilization antigen P25 mediates several functions essential to ookinete survival but is poorly immunogenic in humans. Previous clinical trials targeting this antigen have suggested that conjugation to a carrier protein could improve the immunogenicity of P25. Here we report the production, and characterization of a vaccine candidate composed of a chimeric *P. vivax* Merozoite Surface Protein 1 (cPvMSP1) genetically fused to *P. vivax* P25 (Pvs25) designed to enhance CD4^+^ T cell responses and its assessment in a murine model. We demonstrate that antibodies elicited by immunization with this chimeric protein recognize both the erythrocytic and sexual stages and are able to block the transmission of *P. vivax* field isolates in direct membrane-feeding assays. These findings provide support for the continued development of multi-stage transmission blocking vaccines targeting the life-cycle stage responsible for clinical disease and the sexual-stage development accountable for disease transmission simultaneously.

## Introduction

Malaria remains one of the most serious threats to global health. In 2017, there were an estimated 219 million malaria cases resulting in 435,000 deaths worldwide (World Health Organization, 2018). Of the five *Plasmodium* species that cause malaria in humans, *P. vivax* is the most widely distributed with ~2.8 billion people at risk of infection (Guerra et al., 2010). Its wide geographical range is mainly due to the ability of *P. vivax* to develop within the *Anopheles* mosquito vector at lower temperatures, allowing for its survival at higher altitudes and temperate climates (World Health Organization, 2014). Furthermore, *P. vivax* has the ability to produce hypnozoites, dormant liver-stage parasites present in *P. vivax* but not in *P. falciparum* (Krotoski et al., 1982), causing relapse infections weeks to months after the initial infection. Effective malaria control programs, therefore, require comprehensive measures that involve targeting both *Plasmodium* species (Battle et al., 2012; Gething et al., 2012).

Current malaria control efforts have mainly been focused on the use of vector-based interventions, including long-lasting insecticide-treated nets (LLIN), indoor residual spraying (IRS), and preventative therapies. Preventive therapies include intermittent preventative treatment in pregnancy (IPTp) with sulfadoxine-pyrimethamine and seasonal malaria chemoprevention (SMC) in children aged 3–59 months living in areas of high seasonal malaria transmission (World Health Organization, 2018). While these interventions have resulted in a significant reduction in *P. falciparum* cases (Mendis et al., 2009), *P. falciparum* vector-based interventions are less efficacious against *P. vivax* (Bockarie and Dagoro, 2006). Anomalous climate patterns, as well as the emergence of mosquito resistance to insecticides (Corbel et al., 2007; Dondorp et al., 2009) and parasite resistance to antimalarial treatments (Thomas et al., 2016; Haldar et al., 2018; World Health Organization, 2018), pose additional challenges to the prevention and treatment of malaria despite improved malaria control coverage.

Due to the numerous challenges faced by traditional malaria control methods, the development of novel intervention tools is essential. One potential strategy is the use of transmission-blocking vaccines as they are considered one of the best alternatives to achieve malaria control. Since the life cycle of *Plasmodium* requires the female *Anopheles* mosquito to ingest gametocytes during a blood meal from an infected human host to reach the mosquito midgut and begin the next stage of development outside the human red blood cells, this transition could be interrupted by anti-parasite antibodies present in the blood meal (Saxena et al., 2007).

There are two kinds of transmission blocking antigens that can be targeted by vaccines: pre-fertilization and post-fertilization antigens. Pre-fertilization antigens are expressed by gametocytes and gametes; antibodies against these antigens can block the formation of zygotes by binding to the gametes (Sauerwein and Bousema, 2015). Post-fertilization antigens are expressed by zygotes and ookinetes, antibodies that recognize these forms prevent the mosquito midgut invasion (Saxena et al., 2007; Sauerwein and Bousema, 2015). Under natural conditions, the human host is not exposed to post-fertilization antigens. However, transmission-blocking vaccination can be used to elicit antibodies targeting post-fertilization antigens that the mosquito will be exposed to during the blood meal.

Of the post-fertilization antigens described to date, the P25 protein present on the surface of ookinetes and oocysts, first described by Tsuboi et al. (1998), is one of the best characterized (Blagborough et al., 2016). P25 mediates several functions including promoting the clustering of the ookinetes and allowing them to survive the midgut proteolytic environment (Gass and Yeates, 1979). P25 also mediates the attachment and invasion of the mosquito midgut by damaging the midgut epithelium (Han et al., 2000; Zieler and Dvorak, 2000; Vlachou et al., 2004), and binding to laminin and collagen IV in the basal membrane which serves as the starting signal for the ookinete to oocyst development (Vlachou et al., 2001; Arrighi and Hurd, 2002).

While previous phase I clinical trials using the *P. vivax* P25 protein (Pvs25) have demonstrated that humans can produce antibodies against this antigen, an ideal formulation has not been reported. The first clinical trial of a protein-based Pvs25 vaccine candidate formulated with alum as an adjuvant showed poor immunogenicity and no transmission blocking effect (Malkin et al., 2005). A subsequent clinical trial using a protein-based Pvs25 formulated with Montanide ISA 51 as an adjuvant, showed that low doses of the formulation were able to induce transmission blocking immunity, but higher doses were associated with systemic adverse events (Wu et al., 2008). However, pre-clinical and clinical studies aimed at improving the suboptimal immunogenicity observed by immunization with the *Plasmodium* P25 proteins, Pfs25, and Pvs25 have suggested that the addition of a carrier protein could potentially enhance the immunogenicity of this protein (Qian et al., 2007; Parzych et al., 2017; Radtke et al., 2017).

A vaccine targeting only a transmission-blocking antigen faces challenges in maintaining an antibody response to parasite antigens to which there would be no bosting effect by natural exposure. Furthermore, this type of vaccine would not provide the human host with protection against infection and would likely have low compliance especially if multiple vaccinations are required. We hypothesize that the development of a bifunctional *P. vivax* vaccine able to target both a blood stage antigen and a sexual stage antigen could provide protection against infection to the vaccinated individual as well as reduce transmission. A multi-stage transmission-blocking vaccine is particularly relevant for *P. vivax* given the fact that most relapses are asymptomatic (Van den Eede et al., 2011). These individuals are less likely to receive treatment to clear the infection, resulting in longer periods where the parasite can be transmitted to mosquitoes. In addition to targeting a reservoir of malaria transmission, a *P. vivax* bifunctional blood stage and transmission blocking vaccine may also improve vaccine uptake due to its potential to provide clinical immunity, as well as a reduction in transmission.

Our group has previously defined several CD4^+^ T cell epitopes within the erythrocytic stage antigen Merozoite Surface Protein 1 (MSP1) of *P. vivax*. These epitopes contain features that define them as promiscuous T cell epitopes (i.e., able to bind a broad range of MHC class II alleles) (Caro-Aguilar et al., 2002). Synthetic peptides representing these *P. vivax* MSP1 T cell epitopes are recognized by lymphocytes from individuals naturally infected with *P. vivax* (Caro-Aguilar et al., 2002). We have designed and expressed a chimeric *P. vivax* MSP1 (cPvMSP1) by genetically linking five of these promiscuous T cell epitopes arrayed in tandem conformation to an extended version of the carboxyl-terminal 19kDa fragment of the *P. vivax* MSP1 Merozoite Surface Protein 1 (PvMSP1_19_) (Fonseca et al., 2016). We have shown that immunization with cPvMSP1 induced significantly increased cellular and humoral immune responses in the murine model when compared to the native protein (Fonseca et al., 2016). Here we report the design, production, and characterization of a chimeric bifunctional protein composed of the previously described cPvMSP1 (Fonseca et al., 2016), now genetically fused to recombinant Pvs25 (cPvMSP1-Pvs25). We hypothesize that cPvMSP1 will serve both as a carrier protein that can improve Pvs25 immunogenicity while also inducing robust anti-blood stage protective immune responses. Here we assessed the cellular and humoral immunogenicity of cPvMSP1-Pvs25 in mice and its ability to induce long-lived plasma cells, as well as the ability of antibodies elicited by vaccination with cPvMSP1-Pvs25 to reduce transmission when tested in functional assays.

## Methods

### Ethics Statements

This study including human samples was carried out in accordance with the recommendations of the ICH/GCP guidelines, Comité de Etica para Investigación con Humanos, Centro Internacional de Vacunas (CECIV, Cali, Colombia), and the protocol approved by the CECIV. All subjects gave written informed consent in accordance with the Declaration of Helsinki.

All animal protocols that include experimental animal procedures using mice and NHP were carried out in accordance with the US Animal Welfare Act and approved by the Emory University's Institutional Animal Care and Use Committee and followed accordingly.

### Design and Biochemical Characterization of the *P. vivax* Chimeric Pvs25-MSP1 Protein

**Figure 1:**
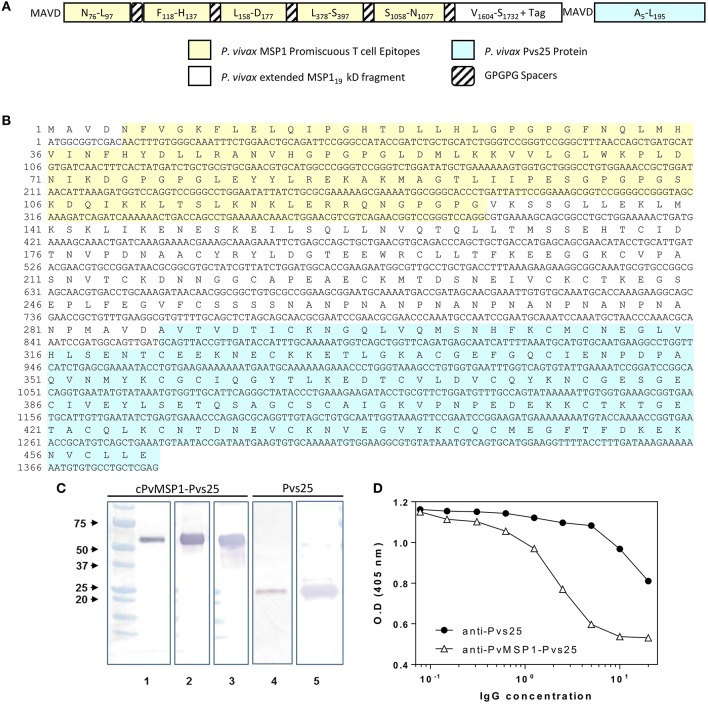
cPvMSP1-Pvs25 protein structure, sequence, and characterization. **(A)** cPvMSP1-Pvs25 structure. This protein includes five promiscuous T cell epitopes of cPvMSP1 (yellow), each separated by a GPGPG spacer (diagonal lines). The promiscuous T cell epitopes are linked to a fragment derived from *P. vivax* MSP1_33_ and the entire *P. vivax* MSP1_19_ kD fragment (white), and the *P. vivax* Pvs25 protein (blue), with each separated by GPGPG spacers (diagonal lines). **(B)** cPvMSP1-Pvs25 amino acid sequence is shown in single letter code. The carboxyl-terminal (H)_6_ tag provided by the vector was not included in the sequence. The yellow shaded area shows the region of the chimeric protein that contains the promiscuous T cell epitopes. The blue shaded area shows the *P. vivax* Pvs25 protein. **(C)** Western blot analysis of the purified cPvMSP1-Pvs25 (lanes 1–3) and the purified Pvs25 proteins (lanes 4 and 5). Each protein was run as separate PAGE gels using 1.0 μg total of purified protein under reducing conditions, and blots were stained with individual antibodies. Full uncut blots are shown in Supplementary Figure 1. Samples were incubated with following antibodies: Lane 1 and 4, the monoclonal antibody N1-1H10 which targets Pvs25; lane 2 and 5, an anti-His-Tag monoclonal antibody targeting the C terminal tags of the cPvMSP1-Pvs25 and Pvs25 proteins; lane 3, the monoclonal antibody 2A10 which targets the cPvMSP1 C terminal tag. The molecular weight markers (BioRad) are indicated. **(D)** Polyclonal anti-cPvMSP1-Pvs25 and anti-Pvs25 elicited in rabbits compete for binding with the transmission blocking monoclonal antibody N1-1H10. Fixed amounts of N1-1H10 (1 μg) were tested with 2-fold dilutions of purified rabbit IgG using Pvs25 as antigen. O.D. values (y-axis) are shown for anti-Pvs25 in closed circles and anti-cPvMSP1-Pvs25 in open triangles using polyclonal antibodies ranging from 0.078 ng/ml to 20 μg/ml (x-axis). Data are presented as geometric mean values.

The 861 bp synthetic gene encoding the chimeric *P. vivax* merozoite surface protein 1 protein (cPvMSP1) used for these studies has been previously described (Fonseca et al., 2016). A 546 bp synthetic gene encoding Pvs25 (codon optimized for expression in *E. coli*) was produced by Geneart (Regensburg, Germany). The sequence for the synthetic gene was derived from the Salvador I strain (XP_001608460; A23 to L195), which does not include its signal peptide and the GPI anchor. A sequence encoding the peptide MAVD was added upstream of the amino-terminal A23 for protein expression. The synthetic gene was subcloned into a pET24d(+) vector. For the production of the synthetic gene encoding the bifunctional chimeric protein, the chimeric PvMSP1 plasmid construct was digested with XhoI and the Pvs25 plasmid amplified by PCR using XhoI/NcoI specific primers. The fragments were annealed and then ligated with T4 DNA ligase. The proper configurations of the *Pvs25* and *cPvMSP1-Pvs25* genes were verified by enzyme restriction analysis and the sequence confirmed using an automatic sequencer. The recombinant pET plasmids were transformed into BL21 (DE3) cells with kanamycin selection. The sequence of the recombinant bifunctional erythrocytic stage-transmission blocking chimeric protein, designated cPvMSP1-Pvs25 (Figure 1A), includes: (i) MAVD amino terminus to reduce degradation during synthesis in *E. coli* and to provide a start signal; (ii) Five promiscuous T cell epitopes derived from *P. vivax* MSP1 capable of binding to a broad range of MHC class II alleles, arranged in tandem interspaced with GPGPG spacers: PvT4 (N_78_-L_97_), PvT6 (F_118_-H_137_), PvT8 (L_158_-D_177_), PvT19 (L_378_-S_397_), and PvT53 (S_1058_-N_1077_); (iii) An extended version of the *P. vivax* MSP1_19_ kD protein fragment, which includes two promiscuous T cell epitopes derived from MSP1_33_ protein fragment; (iv) A (NANP)_6_ tag from the original chimeric PvMSP1 derived from the *P. falciparum* circumsporozoite protein included for biochemical characterization of antigenic integrity of the chimeric protein and to provide an optional affinity purification tag; and (v) The Pvs25 sequence derived from the *P. vivax* Salvador I strain, without the signal peptide and the GPI anchor, but including the MAVD sequence derived from the plasmid Pvs25.

Protein expression was induced with 1 mM IPTG, and the soluble Pvs25 was purified with a Ni-NTA affinity column. cPvMSP1-Pvs25 was expressed in inclusion bodies and refolded as previously described (Singh et al., 2001) using 4M concentration of urea in the refolding solution. After refolding, the protein was purified using gel filtration chromatography. The integrity of the proteins was analyzed by western blot using the anti-Pvs25 monoclonal antibody (mAb) N1-1H10 (MRA-471, BEI Resources), an anti-His tag mAb, or the mAb 2A10 that recognize the (NANP)_6_ carboxyl terminal tag of the cPvMSP1-Pvs25 (Figure 1 and Supplementary Figure 1). Additionally, endotoxin levels of the purified protein product were determined using the E-Toxate *Limulus* amebocyte lysate kit (Sigma), according to the manufacturer's instructions, and were determined to range between 25 and 42 EU/mg of protein.

### Synthetic Peptides

A library of 61 15-mer synthetic peptides overlapped by 11 residues and spanning the complete cPvMSP1-Pvs25 chimeric protein sequence was commercially synthesized by the multiple solid-phase technique (Sigma-Aldrich). Peptide pools were used to characterize cellular reactivity, with the cPvMSP1 peptide pool 1 representing the sequence of the cognate T cell epitopes included in our chimeric construct and the cPvMSP1 pool 2 representing the complete amino acid sequence of the MSP1_19_ kD protein fragment. Pvs25 pool 1 and pool 2 represent the amino acid sequence of Pvs25 (Table 1).

**Table 1:** Chimeric PvMSP1 and Pvs25 peptide pools.

**Peptide pool**	**Sequence**	**Peptide pool**	**Sequence**
cPvMSP1 Pool 1	MANFVGKFLELQIPG VGKFLELQIPGHTDL LELQIPGHTDLLHLG IPGHTDLLHLGPGPG TDLLHLGPGPGFNQL HLGPGPGFNQLMHVI GPGFNQLMHVINFHY NQLMHVINFHYDLLR HVINFHYDLLRANVH FHYDLLRANVHGPGP LLRANVHGPGPGLDM NVHGPGPGLDMLKKV PGPGLDMLKKVVLGL LDMLKKVVLGLWKPL KKVVLGLWKPLDNIK LGLWKPLDNIKDGPG KPLDNIKDGPGPGLE NIKDGPGPGLEYYLR GPGPGLEYYLREKAK GLEYYLREKAKMAGT YLREKAKMAGTLIIP KAKMAGTLIIPESGP AGTLIIPESGPGPGS IIPESGPGPGSKDQI SGPGPGSKDQIKKLT PGSKDQIKKLTSLKN DQIKKLTSLKNKLER KLTSLKNKLERRQNG LKNKLERRQNGPGPG LERRQNGPGPGVKSS	Pvs25 Pool 1	MAVDAVTVDTICKNG AVTVDTICKNGQLVQ DTICKNGQLVQMSNH KNGQLVQMSNHFKCM LVQMSNHFKCMCNEG SNHFKCMCNEGLVHL KCMCNEGLVHLSENT NEGLVHLSENTCEEK VHLSENTCEEKNECK ENTCEEKNECKKETL EEKNECKKETLGKAC ECKKETLGKACGEFG ETLGKACGEFGQCIE KACGEFGQCIENPDP EFGQCIENPDPAQVN CIENPDPAQVNMYKC PDPAQVNMYKCGCIQ QVNMYKCGCIQGYTL YKCGCIQGYTLKEDT CIQGYTLKEDTCVLD
cPvMSP1 Pool 2	QNGPGPGVKSSGLLE GPGVKSSGLLEKLMK KSSGLLEKLMKSKLI LLEKLMKSKLIKENE LMKSKLIKENESKEI KLIKENESKEILSQL ENESKEILSQLLNVQ KEILSQLLNVQTQLL SQLLNVQTQLLTMSS NVQTQLLTMSSEHTC QLLTMSSEHTCIDTN MSSEHTCIDTNVPDN HTCIDTNVPDNAACY DTNVPDNAACYRYLD PDNAACYRYLDGTEE ACYRYLDGTEEWRCL YLDGTEEWRCLLTFK TEEWRCLLTFKEEGG RCLLTFKEEGGKCVP TFKEEGGKCVPASNV EGGKCVPASNVTCKD CVPASNVTCKDNNGG SNVTCKDNNGGCAPE CKDNNGGCAPEAECK NGGCAPEAECKMTDS APEAECKMTDSNEIV ECKMTDSNEIVCKCT TDSNEIVCKCTKEGS EIVCKCTKEGSEPLF KCTKEGSEPLFEGVF EGSEPLFEGVFCSSS	Pvs25 Pool 2	YTLKEDTCVLDVCQY EDTCVLDVCQYKNCG VLDVCQYKNCGESGE CQYKNCGESGECIVE NCGESGECIVEYLSE SGECIVEYLSETQSA IVEYLSETQSAGCSC LSETQSAGCSCAIGK QSAGCSCAIGKVPNP CSCAIGKVPNPEDEK IGKVPNPEDEKKCTK PNPEDEKKCTKTGET DEKKCTKTGETACQL CTKTGETACQLKCNT GETACQLKCNTDNEV CQLKCNTDNEVCKNV CNTDNEVCKNVEGVY NEVCKNVEGVYKCQC KNVEGVYKCQCMEGF GVYKCQCMEGFTFDK CQCMEGFTFDKEKNV EGFTFDKEKNVCLLE

*Peptide pools used for stimulation of T cells for assessment of cytokine production after the second immunization. cPvMSP1 Pool 1 represents MSP1 T cell epitopes present in cPvMSP1. Pool 2 represents the extended MSP1_19_ kD protein fragment. Pvs25 pools 1 and 2 cover the full sequence of the Pvs25 protein*.

### Mice Immunizations

Groups of 10 female CB6F1/J (H-2^d/b^) mice, 6–8 weeks of age, were purchased from The Jackson Laboratory. The animals were immunized subcutaneously on days 0, 20, and 40, in the base of the tail and the interscapular area, using 20 μg of either the cPvMSP1-Pvs25 or Pvs25 proteins emulsified in the adjuvant Montanide ISA 51 VG (Seppic). As a control, a group of mice received PBS emulsified in the same adjuvant. A summary of the immunization regimens and groups can be found in Table 2. All animal protocols were approved by the Emory University's Institutional Animal Care and Use Committee and followed accordingly. Rabbits were immunized four times either with cPvMSP1-Pvs25 or Pvs25 at twenty days intervals, and sera were obtained prior to the first immunization and after each immunization. Rabbit immunization and sera collections were performed by Convance Inc.

**Table 2:** Immunization regimens.

**Regimen**	**Prime day 0**		**Boost day 20**		**Boost day 40**	
	**Protein**	**Dose**	**Protein**	**Dose**	**Protein**	**Dose**
cPvMSP1-Pvs25	cPvMSP1-Pvs25	20 μg	cPvMSP1-Pvs25	20 μg	cPvMSP1-Pvs25	20 μg
Pvs25 protein	Pvs25 protein	20 μg	Pvs25 protein	20 μg	Pvs25 protein	20 μg
Adjuvant Control	Montanide ISA 51 VG		Montanide ISA 51 VG		Montanide ISA 51 VG	

*CB6F1/J mice received subcutaneous immunizations at days 0, 20, and 40 with 20 μg of either cPvMSP1-Pvs25 or Pvs25, both emulsified at a 1:1 volume ratio with the adjuvant Montanide ISA 51 VG. An equivalent volume of PBS was emulsified at a 1:1 volume ratio for subcutaneous injections in the adjuvant control group at the same intervals*.

### ELISA Assays

The procedures for the assessment of IgG antibody titers, subclasses, and avidity have been previously described (Fonseca et al., 2016). Antibody titers elicited by immunization of mice were determined by ELISA using Immulon 4HBX plates (Thermo Scientific) coated with 1 μg/ml of cPvMSP1-Pvs25, Pvs25, or PvMSP1_19_ diluted in carbonate buffer as described (Singh et al., 2010).

Briefly, plates were allowed to incubate overnight with 100 μl of the 1 μg/ml protein solution. The solution was removed, and plates were washed 3 times with wash buffer consisting of PBS 1X with 0.05% Tween 20. 200 μl of blocking solution, BSA (KPL) diluted 1:10 in distilled water, was added to each well and plates were incubated again for 2 h at 37°C. Blocking solution was removed without washing. Sera were diluted in a dilution solution composed of 1:20 BSA (KPL) in distilled water at a starting dilution of 1:320. Cutoffs for positive titers were set at the highest dilution of sera where the O.D. was greater than that of the mean plus three standard deviations above the optical densities obtained using pre-immune sera. Following a 1-h incubation with 100 μl of the diluted mouse sera at 37°C, the plates were washed five times with wash buffer before the addition of 100 μl of peroxidase labeled goat anti-mouse IgG antibody (KPL) at 1:1,000 in dilution solution. Plates were again incubated for 1 h at 37°C before washing five times with wash buffer. ABTS solution (KPL) was used as a substrate following a 1-h incubation. Optical densities were determined using a VERSAmax ELISA reader (Molecular Device Corporation) with a 405 nm filter. Results are presented as the reciprocal of the end-point dilution.

IgG1 and IgG2a subclass profiles of vaccine-induced antibodies were also determined. ELISA assays were performed as described for the determination of antibody titers, except that after incubation with sera the plates were washed and incubated with biotinylated rat anti-mouse mAbs IgG1 or IgG2a, (BD Pharmingen) for 2 h. After washing, the bound antibodies were detected using horseradish peroxidase (HRP)-streptavidin (KPL) and the SureBlue™ TMB Microwell Peroxidase Substrate (KPL). The peroxidase reaction was stopped with the TMB Stop Solution (KPL). Optical densities were determined using a VERSAmax ELISA reader (Molecular Device Corporation) with a 450 nm filter.

The avidity indices of the antibodies were assessed by ammonium thiocyanate elution-based ELISA using sera samples obtained at day 60, corresponding to 20 days after the final immunization, and day 730, (2 years after the first immunization). The avidity ELISA was conducted similarly to the total IgG titer ELISA, with slight modifications. Briefly, serial dilutions of the sera were assayed in the absence and presence of 1M NH_4_SCN (Sigma Aldrich) in PBS. The plates were incubated for 15 min at room temperature before washing and proceeding with the assay as described above. The avidity index was calculated as the ratio between the antilog of the absorbance curves obtained with (*x*_1_) and without (*x*_2_) NH4SCN, as previously described (Perciani et al., 2007).

For ELISA competition assays, purified polyclonal anti-cPvMSP1-Pvs25 and anti-Pvs25 elicited in rabbits (Covance) were used. Fixed amounts of the monoclonal antibody N1-1H10 (1 μg) were tested with 2-fold dilutions of purified rabbit IgG using the recombinant Pvs25 protein as antigen. The concentration of polyclonal antibodies required for 50% inhibition of Pvs25-N1-1H10 interaction was then estimated using linear regression.

### Indirect Immunofluorescence Assays (IFA)

Sera obtained from 10 C5B6F1/J mice after the third immunization with cPvMSP1-Pvs25 were pooled, and antibody reactivity against native *P. vivax* PvMSP1 protein was evaluated by indirect immunofluorescence. For assessment of antibody reactivity, an aliquot of blood was collected from a *P. vivax* infected *Saimiri boliviensis* monkey (kindly provided by Dr. Mary Galinski) into CPD tubes and washed twice using RPMI 1640 medium before the cells were adjusted to 1% hematocrit. Ten microliters of the cell suspension were added to wells of 12-well slides (ICN Biomedicals Inc) and air-dried before storage at −20°C. To evaluate reactivity, parasites slides were air-dried at RT for 30 min. Afterward, slides were incubated 90 min with mouse sera, diluted at 1:500 in PBS with 0.2% BSA in a dark, moist chamber. After the incubation, slides were washed 3 times with PBS containing Tween 20 (PBS-T), to minimize non-specific binding. Parasites were stained for 30 min at RT in a dark, moist chamber with goat anti-mouse Alexa Fluor 488 (Invitrogen) at a 1:500 dilution in Evans Blue 0.4% in PBS 1X. After staining, microscope slides were washed 3 times and allowed to dry completely. Parasite nuclei were stained using 4′,6-diamidino-2-phenylindole dihydrochloride (DAPI) included in the anti-fade mounting medium ProLong Gold (Life Technologies).

For assessment of reactivity to Pvs25, young oocysts were derived *in vitro* as described (Janse et al., 1985) and produced using the *P. berghei* transgenic parasite expressing Pvs25 (MRA-904, pv25DR, BEI Resources). Culture smears were stored at −80°C until need. Slides were allowed to air dry at room temperature for 30 min before being fixed for 10 min in 4% PFA/PBS. Slides were then washed 3 times with PBS 1X and blocked for 1 h in blocking buffer (10% v/v FBS, 1% w/v BSA in PBS). Slides were allowed to incubate overnight with sera from individual rabbits immunized with either Pvs25 or cPvMSP1-Pvs25 at 1:500 in 1% (w/v) BSA in PBS at 4°C in a wet chamber. The following day, slides were washed 3 times in PBS and then incubated with Alexa Fluor 488-conjugated goat anti-rabbit (H+L) IgG (ThermoFisher) at 1:500 in 0.4% Evans Blue in PBS 1X for 60 min. After washing 3 times, slides were allowed to dry completely (8 h) and mounted with ProLong Gold anti-fade reagent with DAPI (Life Technologies).

### ELISpot Assays

Ninety-six-well plates were coated with 5 μg/ml Pvs25, cPvMSP1-Pvs25, and cPvMSP1 and blocked with complete RPMI (10% FBS, 1% penicillin/ streptavidin, 1% HEPES, and 50 μM 2-mercaptoethanol). Bone marrow and splenic cells were then isolated from the CB6FJ/1 mice 2 years post-immunization with Pvs25, cPvMSP1-Pvs25, or the Montanide adjuvant control. To isolate bone marrow cells from the mice, femurs were removed after the mice were euthanized following Emory IACUC approved procedures. Femurs were then placed in complete RPMI, and the ends of the bones were clipped off with sterile surgical scissors. The bone marrow was then flushed from the femur with RPMI using a syringe into a new sterile conical tube. The bone marrow was then passed through the syringe needle several times to resuspend the cells. Similarly, spleens were removed post-mortem and then mashed through a cell strainer using the plunger end of a syringe. Cells were washed and used immediately with no further processing. Cells were serially diluted on prepared plates and incubated for 16 h at 37°C. The plates were then treated with anti-IgG-biotin (Southern Biotechnology) followed by incubation with streptavidin-alkaline phosphatase (Sigma). Plates were then developed with 5-bromo-4-chloro-3-indolylphosphate (Sigma) until spots appeared, and spots counted with CTL ImmunoSpot software. Results were then normalized to adjuvant control mice.

### Flow Cytometry Assays

Flow cytometry analyses of cPvMSP1-Pvs25 or Pvs25 specific T cells were conducted to simultaneously analyze IFN-γ at the single-cell level in T cells derived from splenocytes obtained 5 days after the final boosting immunization. Mice were euthanized according to the Emory IACUC approved protocols and spleens were removed. Spleens were transferred into complete media, composed of DMEM, 1% non-essential amino acids, 2 mM L-glutamine, 5% inactivated FBS, 50 μM 2-mercaptoethanol, 10 mM HEPES, 100 U/ml penicillin, and 100 μg/ml streptomycin. Spleens were then homogenized under sterile conditions, and the homogenized fluid was passed through 200 μm nylon strainer to remove clumps and large pieces of tissue. Red blood cells were lysed using 2 ml of BD PharmLyse buffer incubated with for 3 min before centrifugation at 400 g for 5 min and washing with 5 ml of flow cytometry buffer. Cells were counted and the concentration was adjusted to 10^7^ cells/ml. 100 μl was then placed in individual round bottom tubes. Cells were stimulated for 6 h with peptide pools at 2 μg/ml at 37°C, in the presence of GolgiPlug (BD Biosciences). Cells were then incubated with Live/Dead Aqua Stain (Life Technologies) followed by surface staining with α-CD3 (PerCP Cy5.5), α-CD4 (Alexa Fluor 700), and α-CD8α (APC-Cy7) for 30 min. The cells were then fixed, permeabilized and stained with antibodies against IFN-γ (APC). All the monoclonal antibodies were obtained from BioLegend. Flow cytometry analyses were performed using an LSRII flow cytometer (BD Biosciences), and data were analyzed using FlowJo software version 10.1. The lymphocytes were initially gated on the Live/Dead channel, and then CD3${}_{,}^{+}$ and then CD4^+^ and CD8^+^ populations. Antigen-specific cytokine-secreting T cells were identified within both the CD4^+^ and CD8^+^ populations. The frequency of antigen-specific cytokine-producing cells was determined by subtracting the percentage of cytokine-producing T cells after incubation with medium alone from the percentage of cytokine-producing T cells after incubation with the peptide pools. Samples that did not meet this requirement were set to zero.

### Transmission-Blocking Assays

The transmission-blocking activity of sera derived from rabbits immunized with Pvs25 or cPvMSP1-Pvs25 was measured by direct membrane feeding assays as described elsewhere (Arevalo-Herrera et al., 2005, 2015; Vallejo et al., 2016). Briefly, 150 μl of infected RBCs from *P. vivax* infected patients were washed twice with RPMI 1640 medium (Sigma Aldrich) and diluted in a 150 μl of sera from rabbits that were not heat-inactivated obtained after three immunizations with 20 μg of cPvMSP1-Pvs25 or Pvs25 to feed 100 adults (2–3 day old) *An. albimanus* mosquitoes. Pre-immune sera from the same rabbits were used as a negative control. After 30 min of feeding, unfed mosquitoes were removed from the cages, and fed mosquitoes maintained at 27°C and 80–90% relative humidity. All procedures were performed at 37°C. Seven days after feeding, 30–40 mosquitoes were dissected, midguts were stained with 2% mercurochrome, and the numbers of oocysts per mosquito midgut were counted.

### Statistical Analysis

Statistical analysis and graphs were made using GraphPad Prism 5.0 software (GraphPad Software Inc.). For analysis of the antibody responses, all ELISA titers were log-transformed to conform to the normality and variance requirements of parametric testing and compared using Student's *t*-test for comparison of antibody titers between groups. Student's *t*-test was used for the comparison of antibody avidity between groups. Mann Whitney was used for the comparison of antibody subclass ratios between immunization groups. Differences in the numbers of antibody secreting cells obtained via ELISpot were analyzed using the Mann-Whitney test to compare responses obtained from the Pvs25 and cPvMSP1-Pvs25 immunization groups. Levels of cytokine IFN-γ production obtained from flow cytometry were analyzed using unpaired *t*-tests to compare immunization groups. The transmission-blocking activity of the anti-cPvMSP1-Pvs25 or anti-Pvs25 sera compared against naïve sera or between the groups was analyzed using one-way ANOVA.

## Results

### Design, Expression, and Characterization of the Chimeric Protein cPvMSP1-Pvs25

The chimeric PvMSP1 protein used to create a bifunctional vaccine construct by genetic fusion to the Pvs25 protein has previously been reported by our group (Fonseca et al., 2016). Briefly, the protein consists of five experimentally defined promiscuous T cell epitopes (Caro-Aguilar et al., 2002) derived from several regions along the native *P. vivax* MSP1. These epitopes are arrayed in tandem, interspaced with GPGPG spacers, and genetically fused to an extended form of the PvMSP1_19_ kD fragment that contains two T helper epitopes derived from MSP1_33_ kD fragment. Initial immunogenicity studies showed that this protein was able to elicit robust cytophilic antibody and CD4^+^ and CD8+ T cell responses when delivered either alone as a recombinant protein formulated in water-in-oil emulsion using homologous prime-boost immunization regimens (Fonseca et al., 2016) or via heterologous prime-boost immunization regimens using recombinant adenoviral vectors for priming immunization (Fonseca et al., 2018). To test the feasibility of developing a hybrid vaccine with the potential of inducing blood and mosquito stage-specific immunity, we expressed cPvMSP1-Pvs25 and Pvs25 as recombinant proteins in *E. coli* (Figures 1A,B).

To confirm the biochemical identity of the cPvMSP1-Pvs25 protein, western blot analysis using monoclonal antibodies targeting specific regions of the cPvMSP1 and Pvs25 protein components was conducted (Figure 1C). The monoclonal antibody N1-1H10 was used for assessment of both the cPvMSP1-Pvs25 and recombinant Pvs25 proteins, as it recognizes a conformational-dependent epitope (Hisaeda et al., 2001) present in the second epidermal growth factor-like domain of Pvs25 (Saxena et al., 2006) and is able to inhibit oocyst development in the vector (Ramjanee et al., 2007). Anti-His tag antibodies were used to identify the His-tag present at the C terminal end of the Pvs25 protein segment of the cPvMSP1-Pvs25 protein and the recombinant Pvs25. The monoclonal antibody 2A10 targets the (NANP)_n_ repeat region derived from *P. falciparum* circumsporozoite protein, which is present in the cPvMSP1-Pvs25 protein at the carboxyl-terminal end of the chimeric PvMSP1 protein. We observed recognition of the cPvMSP1-Pvs25 protein as a single band with the expected size of ~52 kDa by the monoclonal antibody targeting the NANP repeats (2A10), the anti-His tag monoclonal antibody, and the monoclonal antibody targeting the Pvs25 protein (N1-1H10). As expected, we also observed binding of the anti-Pvs25 monoclonal antibody (N1-1H10) and the anti-His-tag antibody to the recombinant Pvs25 protein, shown as a ~25 kDa band. These experiments confirm that at least the epitopes of these monoclonal antibodies remained correctly folded.

To determine if the Pvs25 functional domains are conserved within cPvMSP1-Pvs25, we used ELISA competition assays (Figure 1D). Purified polyclonal antibodies elicited in rabbits by immunization with the recombinant proteins were tested at different concentrations and co-incubated with fixed amounts of the monoclonal antibody N1-1H10 (Ramjanee et al., 2007). The polyclonal antibodies inhibited the binding of N1-1H10 with a distinct competition pattern. The concentration of the polyclonal antibodies required for 50% inhibition of antigen-monoclonal antibody interaction was estimated in 32 μg for anti-Pvs25 and 13 μg for anti-cPvMSP1-Pvs25. These results suggest that antibodies elicited by immunization with the protein expressed in *E. coli* have transmission-blocking potential.

### Antibody Response Induced by cPvMSP1-Pvs25

**Figure 2:**
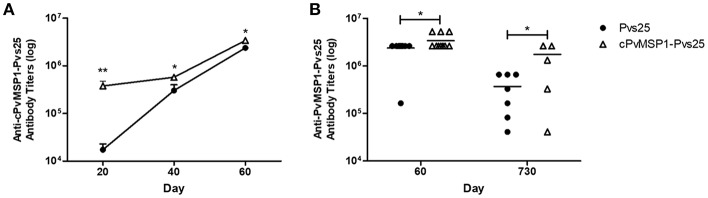
Antibody responses to the chimeric PvMSP1-Pvs25 protein. Antibody responses against the chimeric PvMSP1-Pvs25 protein were assessed in CB6F1/J mice following immunization with either Pvs25 (closed circles) or cPvMSP1-Pvs25 (open triangles). **(A)** Time course of antibody responses to cPvMSP1-Pvs25 on days 20, 40, and 60 (*n* = 10). **(B)** Comparison of antibody titers between day 60 (*n* = 10) and at 2 years (730 days after the first immunization, *n* = 7). The titers against PvMSP1-Pvs25 are shown. Statistical analysis was conducted using Mann Whitney tests. Statistically significant differences are denoted by *p < 0.05 and **p < 0.01.

Hybrid CB6F1/J mice were immunized with Pvs25 or cPvMSP1-Pvs25 on days 0, 20, and 40 (Table 2). Sera were obtained 20 days after each immunization. When we assessed the anti-cPvMSP1-Pvs25 antibody titers at day 20 and 40, we observed significantly higher titers in the cPvMSP1-Pvs25 immunized group than in the Pvs25 group at both time points (*P* = 0.0017 and 0.0262, respectively, Figure 2A). At day 60, 20 days after the last immunization, mice immunized with cPvMSP1-Pvs25 had mean antibody titers of 3.4 × 10^6^ against the chimeric recombinant protein, significantly higher (*P* = 0.0414) than those in mice immunized with Pvs25 which had mean antibody titers of 2.4 × 10^6^ (Figure 2B). At day 730, 2 years after the first immunization, the group immunized with cPvMSP1-Pvs25 had mean antibody titers of 1.7 × 10^6^ against cPvMSP1-Pvs25, a reduction of 26.8%. This observation is in sharp contrast with the group of mice immunized with Pvs25 that had mean antibody titers of 3.7 × 10^5^ against cPvMSP1-Pvs25, a reduction of 89.2% (*P* = 0.0107, Figure 2B). The data suggest that unlike Pvs25 the antibody response induced by cPvMSP1-Pvs25 is long-lasting in mice.

**Figure 3:**
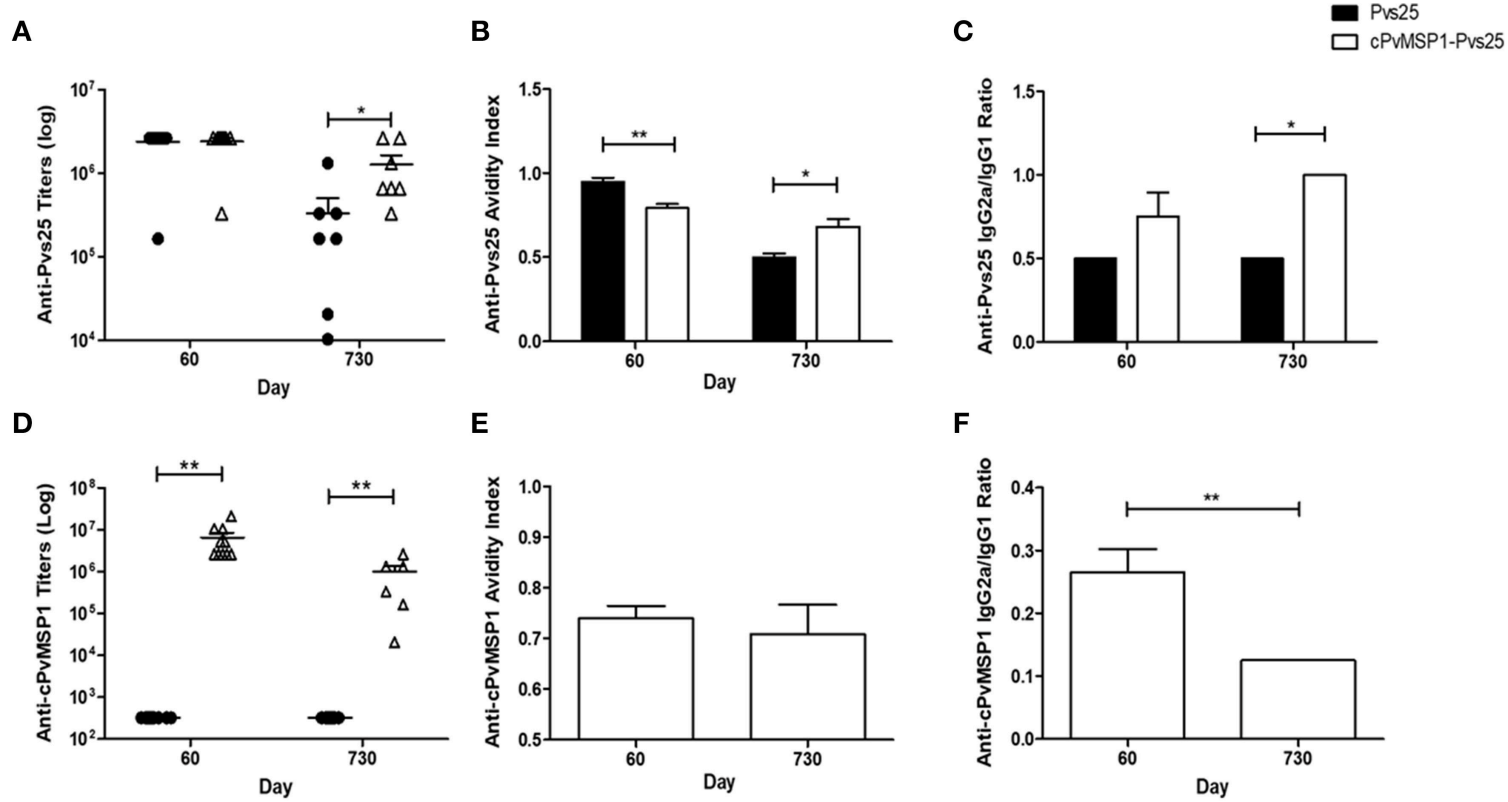
Antibody responses to cPvMSP1 and Pvs25. Antibody responses against the recombinant cPvMSP1 and Pvs25 proteins that form the recombinant cPvMSP1-Pvs25 protein were assessed in CB6F1/J mice following immunization with either Pvs25 (closed circles or bars) or cPvMSP1-Pvs25 (open triangles or bars) on day 60 and 730 after the first immunization. The titers against Pvs25 and cPvMSP1 are shown in **(A,D)**, respectively. Results are presented for *n* = 10 mice for day 60 and *n* = 7 mice for day 730. Avidity indices for anti-Pvs25 and anti-cPvMSP1 are shown in **(B,E)**. IgG subclass responses, displayed as the IgG2a to IgG1 ratio, are shown in **(C)** for Pvs25 and **(F)** for cPvMSP1. Avidity indices and subclass results are presented as the mean values obtained from sera pooled from the 10 mice at day 60 or the 7 surviving mice at day 730, responses were averaged from four technical replicates. Statistical analysis was conducted using unpaired t-tests. Statistically significant differences are denoted by *p < 0.05 and **p < 0.01.

To assess if antigenic competition occurs in mice immunized with the chimeric protein, antibody titers against the individual components of the bifunctional chimeric protein (Pvs25 and cPvMSP1) were also measured (Figure 3). The cPvMSP1-Pvs25 immunization group was able to recognize Pvs25 at day 60 with mean antibody titers of 2.4 × 10^6^, which were similar to levels in mice immunized with Pvs25 (*P* = 0.9617) (Figure 3A). Assessment of anti-Pvs25 titers at day 730 in mice immunized with cPvMSP1-Pvs25 revealed a titers reduction of 47.2%, while the antibody titers in mice immunized with Pvs25 alone were reduced by 86.0%, a significant difference between the groups (*P* = 0.0402) (Figure 3A). As expected, mice immunized with Pvs25 alone did not produce antibodies against PvMSP1, showing only minimal reaction at the highest concentration of sera used. In contrast, mice immunized with cPvMSP1-Pvs25 had mean anti-PvMSP1 antibody titers of 6.6 × 10^6^ at 20 days after the final immunization, and 1.0 × 10^6^ two years later (*P* = 0.0027) (Figure 3D); the later titers at day 730, remained significantly higher than the Pvs25 immunized group, with a reduction of 84.8% (*P* = 0.003).

### Characteristics of Anti-Pvs25 and Anti-MSP1 Antibodies

The average avidity index of anti-Pvs25 antibodies induced by immunization with cPvMSP1-Pvs25 at 20 days after the final immunization was significantly lower than that induced by immunization with Pvs25 (0.79 and 0.95, respectively, *P* = 0.0038) (Figure 3B). In contrast, by day 730 the respective values were 0.68 and 0.50 (*P* = 0.0108), indicating a significantly higher avidity index for antibodies induced by cPvMSP1-Pvs25 compared with Pvs25 alone.

The characteristics of the antibodies against chimeric PvMSP1 were only analyzed in the group immunized with cPvMSP1-Pvs25 since only these mice recognize this antigen. The average avidity index of the cPvMSP1-Pvs25 induced anti-MSP1 antibodies 20 days after the final immunization was 0.74 and remained similar after 2 years (0.71, *P* = 0.6381) (Figure 3E).

The IgG subclasses induced by vaccination with Pvs25 and cPvMSP1-Pvs25 was assessed in order to determine antibody quality, as cytophilic antibodies against MSP1, which correspond to IgG2a in mice, have previously been reported to be associated with protection (Stanisic et al., 2009). Measurement of the Pvs25-specific IgG2a and IgG1 subclasses, expressed as the IgG2a/IgG1 ratio, revealed a higher ratio for the cPvMSP1-Pvs25 immunized mice at both day 60 and day 730, however only at day 730 was the IgG2a/IgG1 subclass ratio of the cPvMSP1-Pvs25 group significantly higher than that of Pvs25 immunized mice (*P* = 0.0131) (Figure 3C). Assessment of the IgG subclass ratios for anti-PvMSP1 responses induced by vaccination with cPvMSP-Pvs25 revealed a different pattern, with the IgG2a/IgG1 ratio of the anti-MSP1 antibodies significantly higher at 60 days than at 730 days after the first immunization (*P* = 0.0010) (Figure 3F), showing a shift toward a Th2 phenotype over time.

### Anti-cPvMSP1-Pvs25 Induced Antibodies Recognize the Native Parasite Proteins

**Figure 4:**
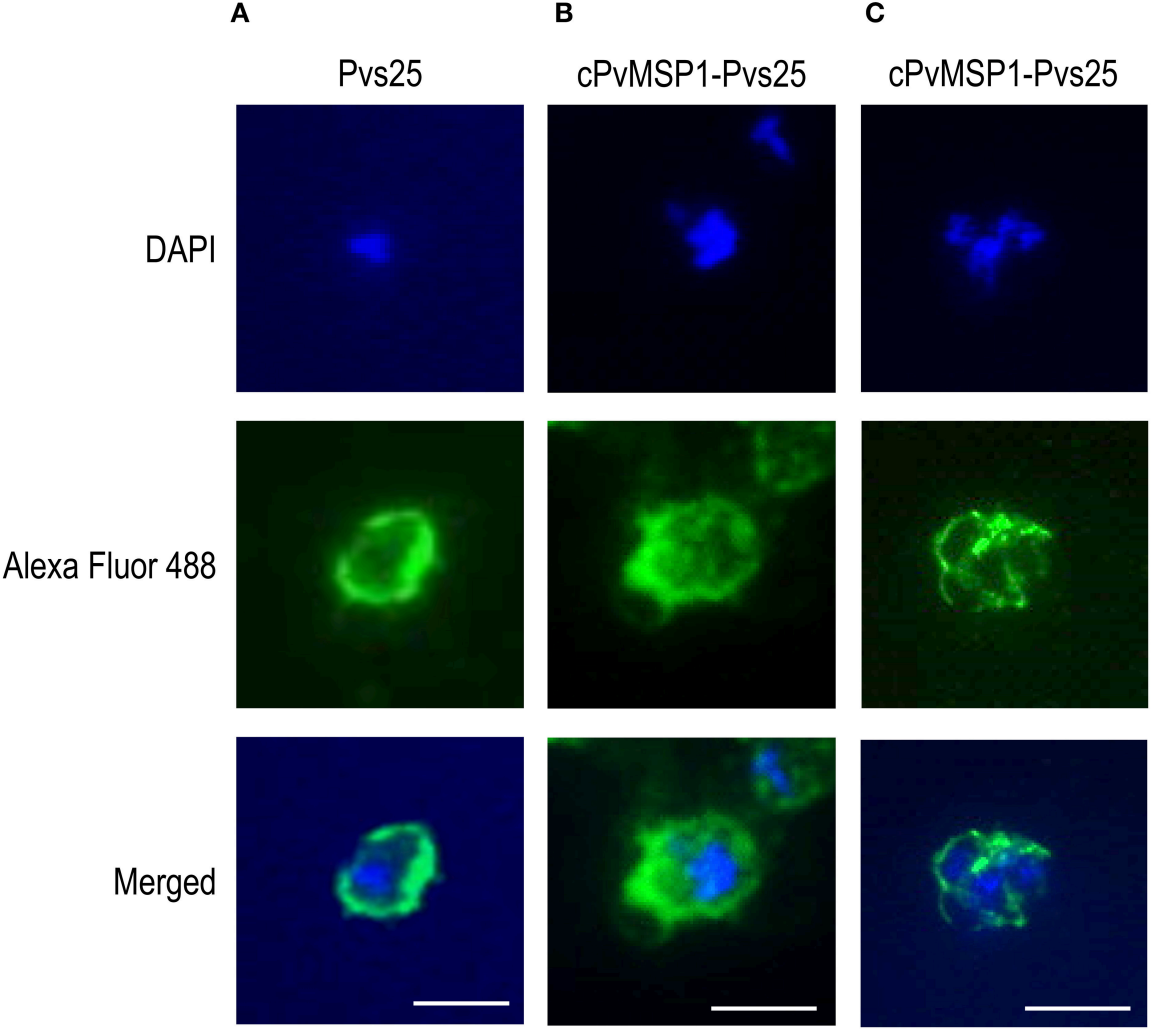
Immunofluorescence assays of young oocysts and schizonts. Sera from individual rabbits immunized with either Pvs25 or cPvMSP1-Pvs25 was used to assess reactivity against *in vitro* derived oocysts using the *P. berghei* transgenic parasite expressing Pvs25 **(A, B)**. A pool of sera obtained from CB6F1/J mice 20 days after the final immunization with cPvMSP1-Pvs25 was used to assess reactivity against blood-stage *P. vivax* parasites **(C)**. The upper panels show staining with DAPI, the middle panels show staining with either goat-anti-rabbit or goat-anti-mouse IgG (H+L) Alexa Fluor 488 secondary antibody, and the bottom panels show the merged images. All images are shown at 100x magnification: scale bars, 5 μm.

The ability of the antibodies induced by vaccination with cPvMSP1-Pvs25 or Pvs25 to recognize the native antigens on the surface of oocysts and schizonts was assessed by immunofluorescence (Figure 4). Transgenic *P. berghei* parasites (MRA-904 parasites) expressing Pvs25 on the surface of young oocysts were used to assess the ability of antibodies from cPvMSP1-Pvs25 or Pvs25-immunized rabbits to recognize native Pvs25. As expected, sera from the cPvMSP1-Pvs25 or Pvs25-immunized rabbits were able to bind to the surface of *P. berghei* Pvs25 transgenic parasites (Figures 4A,B). Thin smears made from infected red blood cells from a *P. vivax* infected *Saimiri* monkey were used to assess the ability of antibodies from cPvMSP1-Pvs25 immunized mice to recognize the native PvMSP1 protein. Pooled sera from the cPvMSP1-Pvs25 immunized group obtained at day 60 recognized *P. vivax* schizonts (Figure 4C). Combined, these data indicate that chimeric cPvMSP1-Pvs25 immunization elicited antibodies capable of binding the native structure of Pvs25 and the native *P. vivax* MSP1.

### cPvMSP1-Pvs25 Plasma Cell Induction

**Figure 5:**
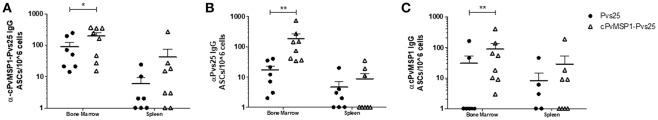
IgG-producing long-lived plasma cells. Antigen-specific IgG plasma cells present in the bone marrow or spleen of CB6F1/J mice immunized with Pvs25 (closed circles, *n* = 7), cPvMSP1-Pvs25 (open triangles, *n* = 8) were analyzed at two years post first immunization. IgG long-lived plasma cells specific to **(A)** cPvMSP1-Pvs25, **(B)** Pvs25, and **(C)** cPvMSP1 are shown. Statistical analysis was conducted using Mann Whitney. Statistically significant differences are denoted by *p < 0.05 and **p < 0.01.

To confirm that the long-lasting antibody responses observed at 2 years post-immunization were related to the induction of long-lived plasma cells (LLPCs), antigen-specific IgG plasma cells were measured in CB6F1/J mice immunized with Pvs25, cPvMSP1-Pvs25, or Montanide via ELISPOT 2 years post-immunization. Mice immunized with cPvMSP1-Pvs25 generated a robust, long-lived IgG plasma cell response not only to the cPvMSP1-Pvs25 protein itself but also to Pvs25 and cPvMSP1 (Figure 5). This response lasts for the lifetime of the animal. The number of long-lived IgG plasma cells in the bone marrow specific to cPvMSP1-Pvs25 was significantly higher (*P* = 0.0456) in mice immunized with cPvMSP1-Pvs25 than those immunized with Pvs25 alone (Figure 5A). Furthermore, the group immunized with Pvs25 was unable to generate a plasma cell response significantly above that of the adjuvant-only control. Mice immunized with cPvMSP1-Pvs25 also generated significantly greater numbers of IgG ASCs specific to Pvs25 (Figure 5B), and PvMSP1 (Figure 5C) compared to mice immunized with Pvs25 (*P* = 0.0022 and *P* = 0.0092, respectively). This increase in long-lived IgG plasma cells was most pronounced in the bone marrow, though antigen-specific IgG plasma cells persist in the spleen at lower numbers. Overall, immunization with cPvMSP1-Pvs25 was more efficient, as it was able to stimulate long-lived, *Plasmodium*-specific IgG plasma cells when compared to standard Pvs25 immunization.

### Cellular Response Induced by cPvMSP1-Pvs25

**Figure 6:**
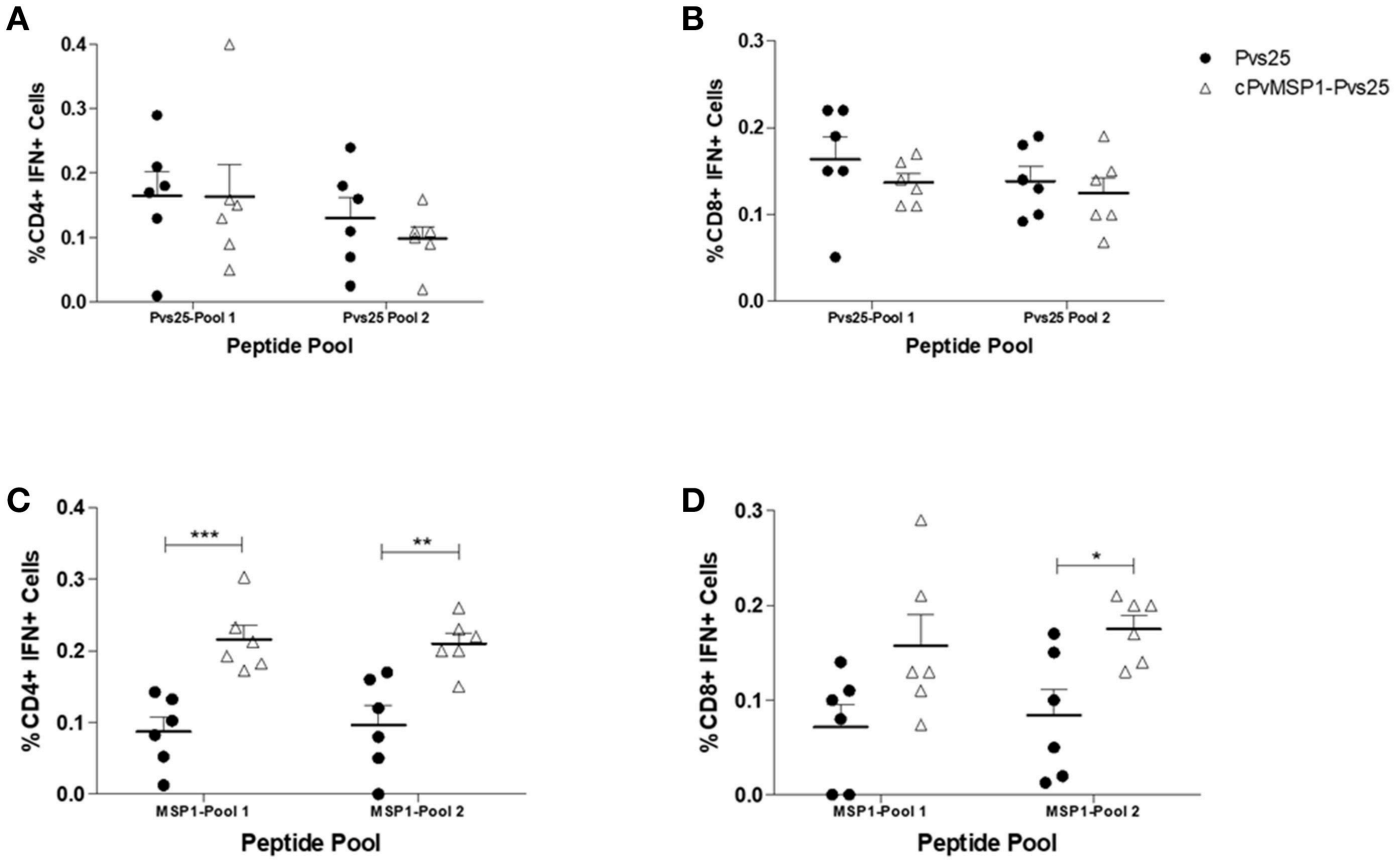
IFN-γ production by CD4^+^ and CD8^+^ T cells following stimulation with Pvs25 and cPvMSP1 peptide pools. Splenocytes obtained from CB6F1/J mice immunized with either Pvs25 (closed circles, *n* = 6) or cPvMSP1-Pvs25 (open triangles, *n* = 6) 5 days after the final immunization were stimulated with peptide pools representing either MSP1 or Pvs25 at 2μg/ml for 2 h. Production of interferon-γ in response to stimulation with Pvs25 peptide pools by **(A)** CD4^+^ T cells and **(B)** CD8^+^ T cells in response to stimulation with cPvMSP1 peptide pools by **(C)** CD4^+^ and **(D)** CD8^+^ T cells are shown. Statistical analysis was conducted using unpaired *t*-tests. Statistically significant differences are denoted by *p < 0.05, **p < 0.01, and ***p < 0.001.

Following the assessment of the humoral response induced by vaccination with cPvMSP1-Pvs25 as compared to Pvs25, we sought to determine if cPvMSP1-Pvs25 was able to induce a cellular response able to recognize individual components of the bifunctional chimeric protein. CB6F1/J mice were immunized 3 times with 20 μg of either the chimeric cPvMSP1-Pvs25 or the recombinant Pvs25 on day 0 and 20, as described in Table 2. Mice were euthanized 5 days after the second immunization and splenocytes were stimulated with peptide pools representing the recombinant Pvs25 and cPvMSP1 proteins (Table 1) to analyze the production of IFN-γ by both CD4^+^ and CD8^+^ T cells (Figure 6). A sample gating strategy is shown in Supplementary Figure 2. We observed no difference in the production of IFN-γ between the immunization groups in either the CD4^+^ or CD8^+^ T cell populations in response to stimulation with the Pvs25 peptide pools (Figures 6A,B). Following stimulation with Pool 1 of cPvMSP1, we found that CD4^+^ T cells from mice immunized with cPvMSP1-Pvs25 produced significantly higher levels of IFN-γ than those immunized with Pvs25 (*P* = 0.0010) (Figure 6C). Similarly, we found that following stimulation with Pool 2 of PvMSP1, both CD4^+^ and CD8^+^ T cells from mice immunized with the cPvMSP1-Pvs25 protein produced significantly more IFN-γ than the Pvs25 immunization group (*P* = 0.0043 for CD4^+^, *P* = 0.0138 for CD8^+^, Figures 6C,D).

### Transmission-Blocking Activity of Anti-cPvMSP1-Pvs25 Antibodies

Sera samples, obtained from rabbits after three immunizations with either cPvMSP1-Pvs25 or Pvs25, were tested for transmission-blocking activity using three different *P. vivax* isolates in independent direct membrane feeding assays. The transmission blocking activity and the number of oocysts counted were assessed for both groups (Table 3). The activity of immune sera was compared to pre-immune control sera (Table 3). Upon dissection of mosquitoes, we found that sera obtained from both Pvs25 and cPvMSP1-Pvs25 resulted in significantly lower percentages of infected mosquitoes when compared to the pre-immune sera samples. Similarly, we found significantly lower numbers of oocysts present in the mosquitoes that had fed on the Pvs25 and cPvMSP1-Pvs25 samples as compared to the pre-immune rabbit sera controls. Overall, our results indicate that antibodies induced by cPvMSP1-Pvs25 are able to block the infection in 90% of exposed mosquitos, and in infected mosquitos, the parasite load is 98% lower than in mosquitoes feeding on a non-immunized source.

**Table 3:** Transmission-blocking activity.

			**Experiment 1**			**Experiment 2**			**Experiment 3**			
**Group**	**Sample**	**Antibody titers by ELISA**	**No. infected (total dissected)^**a**^**	**Average number of oocyst^**b**^**	**TBA (%)^**c**^**	**No. infected (total dissected)^**a**^**	**Average number of oocyst^**b**^**	**TBA (%)^**c**^**	**No. infected (total dissected)^**a**^**	**Average number of oocyst^**b**^**	**TBA (%)^**c**^**	***P*-value^**e**^**
cPvMSP1-Pvs25	Control PI 1	640	20 (36)	155 ± 7.8	85.0	23 (32)	299 ± 13	65.2	10 (30)	29 ± 2.9	100	0.01
	Immune sera 1	1310720	3 (40)	26 ± 6.8		8 (32)	13 ± 1.6		7 (30)	0		
	Control PI 2	640	17 (40)	115 ± 6.8	88.2	23 (30)	262 ± 11.4	69.6	12 (30)	35 ± 2.9	100	0.01
	Immune sera 2	1310720	6 (33)	2 ± 1		7 (30)	29 ± 4.14		0 (30)	0		
Pvs25	Control PI 1	1280	23 (40)	175 ± 7.6	100	19 (30)	262 ± 13.7	63.2	11 (30)	30 ± 2.72	100	0.01
	Immune sera 1	1310720	0 (40)	0		7 (30)	34 ± 4.8		0 (30)	0		
	Immune sera 2^d^	1310720	3 (30)	3 ± 1	87.0	3 (30)	8 ± 2.5	84.2	0 (30)	0	100	0.01
Negative control	AB human serum	NA	20 (36)	657 ± 17.8	NA	23 (32)	413 ± 16.5	NA	18 (30)	320 ± 10.5	NA	

*NA, not applicable*.

a *Number of mosquitoes infected (total number of mosquitoes dissected)*.

b *Average calculated as the total of oocyst /total of mosquitoes dissected ± standard deviation*.

c *Percent inhibition of mean mosquitoes with rabbit sera post-third immunization compared to pre-immune rabbit sera in each independent assay calculated as (1 – [mean mosquitoes in normal rabbit sera/mean mosquitoes post 3rd immunization sera] x 100). A pool of AB normal human sera was used as a negative control*.

d *Pre-immune serum from this rabbit was not tested for TBA, antibody titer was 1280*.

e *Statistical significance P < 0.05*.

*Sera samples from immunized rabbits (cPvMSP1-Pvs25 and Pvs25) were tested in direct membrane-feeding assays (three different experiments) using blood samples collected from naturally infected patients as described (Vallejo et al., 2016)*.

## Discussion

Development of novel malaria intervention tools, such as transmission-blocking vaccines (TBVs), is essential to address the increased reports of insecticide and drug resistance (Corbel et al., 2007; Dondorp et al., 2009), as they could increase the efficiency and sustainability of these existing malaria control methods (Sauerwein, 2007). TBVs rely on the generation of antibodies that block the development of *Plasmodium* parasites within the mosquito midgut. We propose that the ideal TBV formulation should consist of a transmission-blocking component combined with a prophylactic vaccine in order to simultaneously provide protection against disease to the recipient and reduce transmission. Under natural conditions, the human host is not exposed to the post-fertilization antigens. Since there would not be a natural boosting effect during infection, a vaccine targeting a post-fertilization antigen should be highly immunogenic to produce effective antibody responses (Saul, 2007). Here we present evidence that a chimeric protein designed based on one of the best-characterized post-fertilization antigens, P25, conjugated to a chimeric *P. vivax* MSP1 (cPvMSP1-Pvs25), elicits long-lasting antibody responses against both proteins, without immune interference, while inducing robust cellular responses to PvMSP1 in CB6F1/J, a F1 hybrid of BALB/c, and C57BL/6 mice, comparable to those we have previously reported for cPvMSP1 alone in the parental strains (Fonseca et al., 2016).

Assessment of the quality and longevity of the antibody response induced revealed that immunization with cPvMSP1-Pvs25, as compared to Pvs25, resulted in the induction of an antibody response against the immunogens as well as against the individual components, which lasted for the lifetime of the animal with a much lower reduction in antibody titers at 2 years post-immunization. Most critically, we observed a lower reduction in anti-Pvs25 antibody titers at 2 years post-immunization in groups of mice immunized with the bifunctional chimeric protein as compared to the Pvs25 group. These results are significant as an ideal transmission-blocking vaccine candidate must be capable of inducing functional and durable antibodies using a simplified immunization regimen for use in mass administration (Nunes et al., 2014).

For the proof-of-concept studies reported here, we selected Montanide ISA 51 VG, a water-in-oil based adjuvant (Aucouturier et al., 2002) that has been tested before in clinical trials (Baumgaertner et al., 2016; Caballero et al., 2017; Saavedra and Crombet, 2017). Our results are consistent with the reported high efficiency of this adjuvant to induce robust antibody responses given its ability to promote polarization of naïve T cells into T follicular helper cells (Riteau et al., 2016). However, safety concerns have arisen from a Phase I clinical trial of Pfs25 and Pvs25 formulated in Montanide 51 showing systemic adverse events compatible with erythema nodosum (Wu et al., 2008). Recent comparisons of *P. falciparum* Pfs25 formulated in novel oil-in-water based adjuvants compared with alum-based adjuvants found that oil-in-water-based adjuvants EM081 and EM082 were more efficient in eliciting high titers of anti-Pfs25 IgG antibodies than the alum adjuvants (Patra et al., 2015). Interestingly, this study also found that an adjuvant composed of oil-in-water-glucopyranosyl lipid A-induced high-affinity antibodies that effectively blocked infection of mosquitoes with *P. falciparum* and demonstrated that avidity could provide a surrogate measure of efficacy beyond the antibody titers (Patra et al., 2015). The strong adjuvant effect was also confirmed by a Phase I clinical trial of a Pfs25 vaccine candidate paired with the aluminum adjuvant Alhydrogel, which observed a rapid decline in antibody responses after vaccination (Talaat et al., 2016). Overall, these experiments corroborate the adjuvant-dependency on the magnitude and durability of the antibody response of P25-based vaccines (Mlambo et al., 2008; Radtke et al., 2017). The long-lasting immunity induced by cPvMSP1-Pvs25 is therefore very encouraging compared to previous studies and warrant further investigation regarding the best adjuvant system to deliver the bifunctional chimeric protein.

Due to the importance of long-lived plasma cells (LLPC) in maintaining protective antibody levels for years (Radtke et al., 2017), LLPC induced by vaccination with cPvMSP1-Pvs25 were assessed. We were able to confirm high levels of bone marrow and spleen LLPCs at 2 years post-immunization. We observed that immunization with cPvMSP1-Pvs25 was more efficient at generating long-lived *Plasmodium*-specific IgG plasma cells when compared to Pvs25, with significantly more Pvs25-specific LLPCs also observed in the bone marrow. This is in contrast with the poor immunogenicity reported for the native P25 (Tomas et al., 2001; Radtke et al., 2017). Chemical conjugation to carrier proteins, a strategy used for glycoconjugate vaccines to enhance the immunogenicity of bacterial polysaccharides (Rappuoli, 2018), has been tested to improve the immune responses induced by post-fertilization antigens. Clinical trials of the conjugated Pfs25-exoprotein A (EPA) vaccine showed excellent safety profile, but the antibody responses induced by immunization were short lasting with poor responses elicited in volunteers living in endemic areas compared to non-endemic areas (Talaat et al., 2016; Sagara et al., 2018). More recently, chemical conjugation of Pfs25 to tetanus toxoid (TT), a carrier protein used in existing polysaccharide vaccines, resulted in a significant improvement in immunogenicity and longevity (Radtke et al., 2017). TT contains CD4+ T cell epitopes that enhance humoral immunity compensating for the lack of CD4 T cells epitopes within Pfs25 resulting in the generation of T follicular helper (Tfh) cells (Radtke et al., 2017). Tfh cells are required for germinal center B cell differentiation into LLPC essential for long-lasting protection. Similar findings were subsequently reported by Parzych et al. with the *P. falciparum* merozoite surface protein 8 (MSP8) tested as a carrier to deliver Pfs25 (Parzych et al., 2017). These reports highlighted the relevance of T cell epitopes present in the carrier proteins to promote robust immune responses. The likely mechanism of action of the cPvMSP1 within cPvMSP1-Pvs25 is to serve as a carrier protein to the recruitment of *Plasmodium*-specific CD4+ T cells, which ultimately promote a robust humoral immune response to the hybrid vaccine and induction of the anti-Pvs25 LLPC response.

Avidity of the antibodies induced by vaccination with either Pvs25 or cPvMSP1-Pvs25 was also assessed as a measure of immunogenicity. At 20 days after the final immunization, anti-Pvs25 antibody avidity indices were 0.95 for Pvs25 and 0.79 for cPvMSP1-Pvs25 immunization groups. By 2 years post-immunization, the anti-Pvs25 avidity index of the Pvs25 group had fallen to 0.50, significantly lower than the avidity index of 0.68 at 2 years induced by immunization with cPvMSP1-Pvs25. Consistent with the maintenance of antibody avidity against Pvs25 by the cPvMSP1-Pvs25 immunization regimen, the avidity indices of the anti-cPvMSP1 response were not significantly different at 60 days than at 2 years post-immunization. The high avidity antibodies elicited by cPvMSP1-Pvs25 are, therefore, encouraging given their relevance as a biomarker of efficacy of TBVs beyond the antibody titers (Patra et al., 2015). We can also conclude that this outcome is likely an effect of the improved LLPC response induced by the cPvMSP1-Pvs25 vaccination regimen, allowing for the maintenance of higher avidity antibodies over time.

Cytophilic IgG antibody subclass responses directed against MSP1_19_, as well as other merozoite surface antigens, have previously been found to be associated with control of parasitemia and protection from symptomatic illness in children in *P. falciparum* endemic areas (Stanisic et al., 2009). The cytophilic antibody subclasses correspond to IgG1 and IgG3 in humans and IgG2a in mice. We observed a shift in the IgG2a/IgG1 ratio of the anti-cPvMSP1 antibody response between day 60 and 2 years post-prime, with a significant reduction in the IgG2a subclass response. In contrast, there was a significantly higher IgG2a/IgG1 subclass ratio for anti-Pvs25 antibodies in cPvMSP1-Pvs25 immunized mice compared to Pvs25 immunized mice at the 2-year time point. Although we observed higher levels of IgG1 than IgG2a in the anti-cPvMSP1 response, the significantly higher IgG2a/IgG1 ratio observed for the anti-Pvs25 response elicited by cPvMSP1-Pvs25 vaccination is promising and may have biological significance due to differences in the ability of IgG subclasses to activate the classical complement pathway. Parasites present in the mosquito midgut after the female *Anopheles* mosquito ingests infected red blood cells are exposed to multiple plasma elements present in the mosquito blood meal including complement, granulocytes, and the host-derived antibodies (Sauerwein, 2007; Saul, 2007). These components can all affect parasite development within the mosquito and ultimately reduce transmission. Studies have shown that human sera with anti-Pvs25 antibodies have reduced killing activity after heat inactivation indicating that complement may be necessary to block transmission with Pvs25 vaccine-induced antibodies (Malkin et al., 2005). The classical complement pathway involves the binding of complement molecule C1q to the Fc region of the antigen bound antibody. However, IgG subclasses differ in their ability to activate complement, as human cytophilic antibodies IgG3 and IgG1 have been found to more effectively activate the classical complement pathway than the IgG2 subclass. This study suggests that cPvMSP1-Pvs25 immunization is more effective at inducing cytophilic IgG2a subclass antibodies in mice.

The ability of antibodies induced by individual components of the bifunctional chimeric protein in this study to bind the native structures expressed by young oocysts and schizonts is essential for their functional activities. We confirmed by IFAs that antibodies induced by immunization with cPvMSP1-Pvs25 recognized the native structure on the surface of transgenic oocysts expressing Pvs25 as well as the native structure of PvMSP1 in *P. vivax* blood stage schizonts. We have previously shown that passive immunization using antibodies elicited by the orthologous *P. yoelii* chimeric MSP1 protein, based on sequences from the *P. yoelii* 17X strain, protect naïve mice against heterologous challenge with the *P. yoelii nigeriensis* N67 isolates (Singh et al., 2010). Although we were unable to repeat similar passive transfer experiments in this study, future studies using transgenic parasites expressing *P. vivax* MSP1 are warranted. Additionally, the binding capability of anti-cPvMSP1-Pvs25 antibodies and the robust transmission blocking activity elicited by immunization as determined by direct membrane feeding assays, support further studies on the functionality of the anti-Pvs25 and anti-cPvMSP1 antibodies induced by vaccination with cPvMSP1-Pvs25. Furthermore, while no differences in the IFN-γ response were observed between the immunization groups following stimulation with the Pvs25 peptide pools, as expected, stimulation with cPvMSP1 peptide pools revealed that cPvMSP1-Pvs25 induced high levels of IFN-γ CD4^+^ and CD8^+^ T cells as compared to Pvs25-immunized mice. The ability of cPvMSP1-Pvs25 to maintain robust IFN-γ responses to cPvMSP1 when conjugated to the Pvs25 protein is encouraging for the blood stage component of this vaccine candidate, as several reports have indicated that high frequencies of IFN-γ secreting CD4^+^ T cells provide protection from malaria in humans (Roestenberg et al., 2009; White et al., 2013; King and Lamb, 2015).

Direct membrane feeding assays allow the assessment of transmission-blocking activity to determine the functionality of antibodies elicited by vaccination (Bompard et al., 2017). Currently, transmission-blocking assays are considered the most epidemiologically important method of evaluating transmission blocking vaccine candidates (Bompard et al., 2017). We found that sera obtained from both Pvs25 and cPvMSP1-Pvs25-immunized rabbits had significantly high transmission blocking activity, as shown by the lower percentages of infected mosquitoes and the number of oocysts compared with pre-immune sera. Unlike *P. falciparum* membrane feeding experiments, which can be done from parasite cultures, these results are promising as they came from wild *P. vivax* isolates, allowing for better prediction of the transmission blocking potential these antibodies might achieve if tested in clinical trials in endemic areas. These results support further development of cPvMSP1-Pvs25 as an effective transmission-blocking vaccine.

The development of licensed vaccines that reduce malaria transmission of *P. falciparum* and *P. vivax* is one of two main strategic goals of the 2030 Strategic Goals of Malaria Vaccine Technology published by the WHO; the other is a malaria vaccine with at least 75% efficacy against clinical malaria (Moorthy et al., 2013). While there is controversy concerning the use of transmission-blocking vaccines, including the fact that transmission-blocking antigens alone will only reduce transmission but will not protect vaccinated populations from disease (Jamrozik et al., 2015), the combination of transmission blocking antigens with antigens targeting the clinical stages of malaria could be used to prevent both disease and transmission. Studies evaluating the impact of vector control measures have demonstrated that the reduction of transmission in medium to high transmission areas induces a decrease in all-cause mortality, with the youngest age groups of the population benefiting the most (Smith et al., 2001; Rowe and Steketee, 2007; Larsen et al., 2011). We report here that the addition of an anti-erythrocytic stage antigen in the form of the chimeric PvMSP1 protein genetically conjugated to Pvs25 improves the immunogenicity of the TBV candidate while preserving the functionality of Pvs25 induced antibodies. Our results provide support for the continued development of cPvMSP1-Pvs25 and other multi-stage malaria vaccine candidates to address the need for effective vaccines targeting both the parasite stage responsible for the clinical manifestations and simultaneously the sexual stage responsible of transmission.

## Author Contributions

AM conceived the study. JF, BS, JJ, MA-H, and AM designed the experiments. JM, JF, BS, MA-H, MC-M, and CB performed the experiments. JM, JF, and AM performed data analysis. JM, JF, and AM wrote the manuscript. All the authors approved the final version of the manuscript.

### Conflict of Interest Statement

The authors declare that the research was conducted in the absence of any commercial or financial relationships that could be construed as a potential conflict of interest.

## Supplementary Material

Supplementary Figure 1 A full scan of the entire original Western blot analysis of the purified cPvMSP1-Pvs25 **(left)** and the purified Pvs25 proteins **(right)**. Samples were incubated with the specified antibodies: monoclonal antibody N1-1H10 which targets Pvs25; anti-His-Tag monoclonal antibody targeting the C terminal tags of the cPvMSP1-Pvs25 and Pvs25 proteins; or the monoclonal antibody 2A10 which targets the cPvMSP1 C terminal tag. The molecular weight markers (BioRad) are included.

Supplementary Figure 2 Gating strategy for CD4^+^ and CD8^+^ IFN-γ-producing murine T cells. The gating strategy for identifying IFN-γ CD4^+^ and CD8^+^ T cells from murine splenocytes isolated 5 days post boosting includes: FSC singlets, viability gating, CD3^+^ cells, CD4^+^ vs. CD8^+^ T cells, and IFNγ^+^ cells. Data shown represent IFN-γ gating in CD4^+^ T cells of mice receiving Pvs25 or cPvMSP1-Pvs25.
