# Expression of concern: A multi-stage plasmodium vivax malaria vaccine candidate able to induce long-lived antibody responses against blood stage parasites and robust transmission-blocking activity

**DOI:** 10.3389/fcimb.2023.1292315

**Published:** 2023-09-20

**Authors:** Frontiers Editorial Office

With this notice, Frontiers states its awareness of serious allegations surrounding the institutional review board Centro Internacional de Vacunas cited in this article. These allegations are being investigated in line with COPE guidelines. The situation will be updated as soon as the investigation is complete.

