# Supplementary figures and images for: A Multi-Stage Plasmodium vivax Malaria Vaccine Candidate Able to Induce Long-Lived Antibody Responses Against Blood Stage Parasites and Robust Transmission-Blocking Activity
Source: Front Cell Infect Microbiol. 2019 May 1;9:135. doi: 10.3389/fcimb.2019.00135 (PMC6504793; doi:10.3389/fcimb.2019.00135)

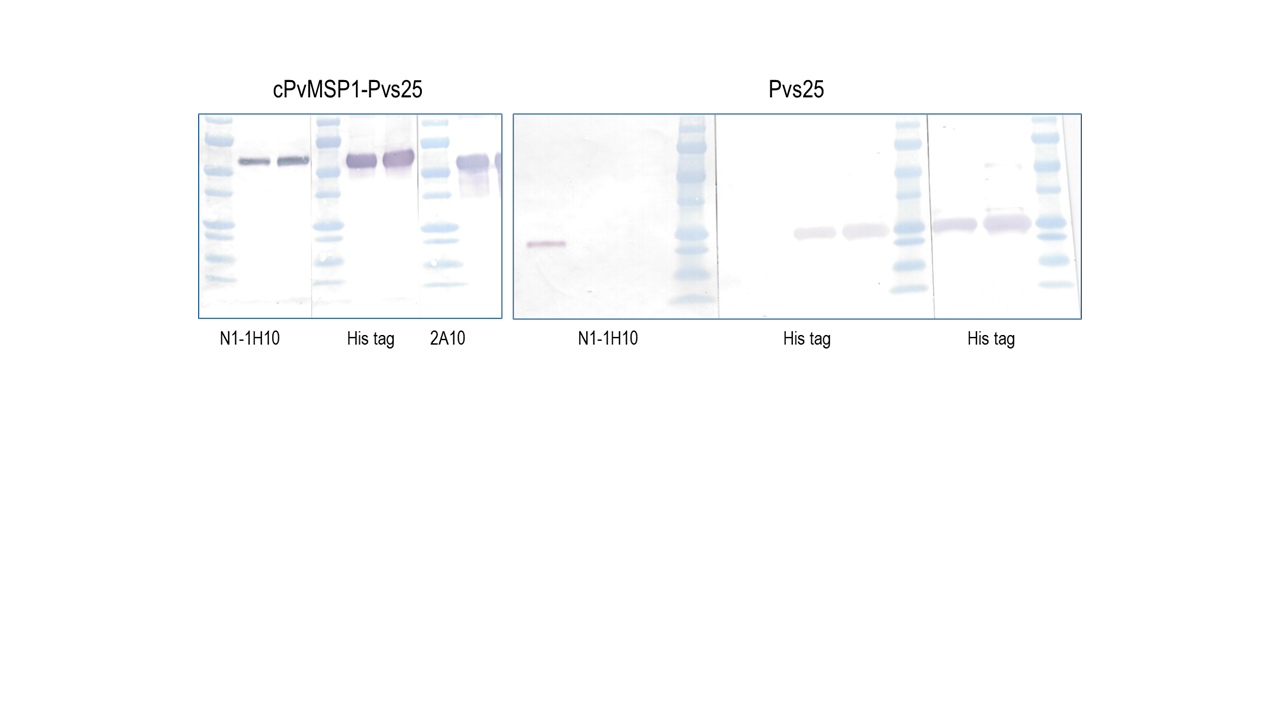

Supplement: Supplementary Figure 1 — A full scan of the entire original Western blot analysis of the purified cPvMSP1-Pvs25 (left) and the purified Pvs25 proteins (right). Samples were incubated with the specified antibodies: monoclonal antibody N1-1H10 which targets Pvs25; anti-His-Tag monoclonal antibody targeting the C terminal tags of the cPvMSP1-Pvs25 and Pvs25 proteins; or the monoclonal antibody 2A10 which targets the cPvMSP1 C terminal tag. The molecular weight markers (BioRad) are included. [file Image_1.TIF]

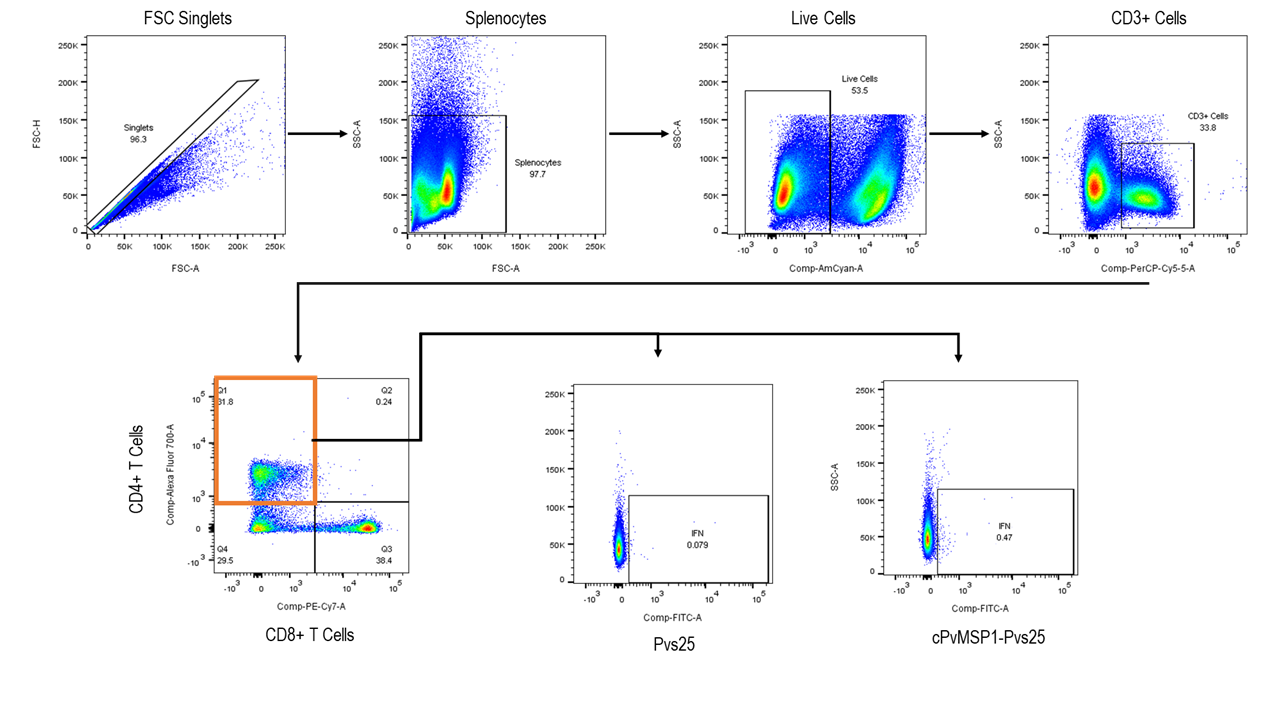

Supplement: Supplementary Figure 2 — Gating strategy for CD4+ and CD8+ IFN-γ-producing murine T cells. The gating strategy for identifying IFN-γ CD4+ and CD8+ T cells from murine splenocytes isolated 5 days post boosting includes: FSC singlets, viability gating, CD3+ cells, CD4+ vs. CD8+ T cells, and IFNγ+ cells. Data shown represent IFN-γ gating in CD4+ T cells of mice receiving Pvs25 or cPvMSP1-Pvs25. [file Image_2.TIF]
